# Biological Advantages of Calcium Hypochlorite Solution Over Sodium Hypochlorite for Regenerative Endodontic Procedures: An Ex Vivo and In Vitro Study on Human Apical Papilla

**DOI:** 10.1111/iej.70038

**Published:** 2025-09-25

**Authors:** Hernán Coaguila‐Llerena, Ellen Rabelo Ferraz, Bárbara Roma Mendes, Luana Raphael da Silva, Carlos Rossa Júnior, Paulo Sérgio Cerri, Gisele Faria

**Affiliations:** ^1^ School of Dentistry, Araraquara, Department of Restorative Dentistry São Paulo State University (UNESP) São Paulo Brazil; ^2^ Department of Endodontics Cayetano Heredia Peruvian University (UPCH) Lima Peru; ^3^ School of Dentistry, Araraquara, Department of Oral Diagnosis and Surgery São Paulo State University (UNESP) São Paulo Brazil; ^4^ School of Dentistry, Araraquara, Department of Morphology and Paediatric Dentistry, Laboratory of Histology and Embryology São Paulo State University (UNESP) São Paulo Brazil

**Keywords:** calcium hypochlorite, dental papilla, regenerative endodontics, sodium hypochlorite

## Abstract

**Aim:**

Calcium hypochlorite [Ca(OCl)_2_] has been proposed as an alternative to sodium hypochlorite (NaOCl) for use as an irrigant. This study aimed to assess morphological changes in human apical papilla (AP) ex vivo, and to evaluate viability, proliferation, chemotaxis and osteogenic differentiation of human apical papilla cells (hAPCs) in vitro following exposure to Ca(OCl)_2_, in comparison to NaOCl.

**Methodology:**

Ex vivo, three AP samples per group were exposed to 1.5% Ca(OCl)_2_, 1.5% NaOCl or control solutions [17% ethylenediaminetetraacetic acid (EDTA) and saline] for 3 min. The AP sections were stained with haematoxylin and eosin (H&E), Masson's trichrome and Alcian Blue for morphological analysis. In vitro, hAPCs were exposed to Ca(OCl)_2_, NaOCl, EDTA or culture medium. Cell viability was assessed with the methyl‐thiazole‐tetrazolium (MTT) assay; proliferation by bromodeoxyuridine incorporation; chemotaxis by transwell assay; and mineralised nodule formation by alizarin red staining. Data were analysed by two‐way ANOVA followed by Tukey's test or by the Kruskal–Wallis and Dunn tests (α = 0.05).

**Results:**

Ex vivo, marked loss of both cells and extracellular matrix components was observed in the outer layer of AP samples, particularly in the NaOCl and Ca(OCl)_2_ groups, with more severe damage found in the NaOCl samples. Samples treated with EDTA exhibited structural organisation similar to those treated with saline. In vitro, Ca(OCl)_2_ induced less cytotoxicity, resulted in the highest proliferation (*p* < 0.05), but promoted lower chemotaxis than the other irrigants (*p* < 0.05). EDTA and Ca(OCl)_2_ led to greater mineralised nodule formation than the other solutions (*p* < 0.05).

**Conclusions:**

Ca(OCl)_2_ at 1.5% caused less structural damage to AP than NaOCl at the same concentration and had a more favourable influence on the viability, proliferation and osteogenic differentiation of hAPCs. Moreover, it did not impair cell chemotaxis. These findings suggest that Ca(OCl)_2_ may offer biological advantages in regenerative endodontic procedures.

## 1 Introduction

Sodium hypochlorite (NaOCl) has long been regarded as the gold standard for endodontic irrigation (Coaguila‐Llerena, Ochoa‐Rodríguez, et al. 2024; Coaguila‐Llerena, Raphael da Silva, and Faria 2024) and has also been employed in regenerative endodontic procedures (REPs) (Galler, Krastl, et al. 2016). However, when extruded into periapical tissues, NaOCl causes severe irritation (Guivarc'h et al. 2017). The cytotoxicity of NaOCl compromises the differentiation of mesenchymal stem cells from the apical papilla (AP) (Martin et al. 2014) and results in dose‐dependent tissue dissolution (Claudino Ribeiro et al. 2022).

Calcium hypochlorite [Ca(OCl)_2_] has been proposed as an alternative to NaOCl (Dutta and Saunders 2012; Coaguila‐Llerena et al. 2022), since it exhibits similar antimicrobial efficacy (Dal Bello et al. 2019) and lower cytotoxicity (Coaguila‐Llerena, Ochoa‐Rodríguez, et al. 2024). Ca(OCl)_2_ is commercially available in powdered granules and, when dissolved in water, releases hypochlorous acid and calcium hydroxide (Dutta and Saunders 2012). A previous study demonstrated that Ca(OCl)_2_ also releases calcium ions, suggesting a potential to promote mineralisation (Coaguila‐Llerena et al. 2022)—that is, the repair of structures following endodontic treatment. This capacity may be particularly relevant in teeth with pulp necrosis and incomplete root formation, in which REPs are indicated.

REPs promote healing of periapical tissues and continuation of root development (Glynis et al. 2021), processes that are mediated by the human apical papilla cells (hAPCs) (Huang et al. 2008). Regeneration involves migration, proliferation and differentiation of mesenchymal stem cells derived from the AP into various cell types responsible for producing mineralised and non‐mineralised tissues, including dental pulp, dentine, cementum and periodontal ligament (Thammajak et al. 2021; Cassiano et al. 2022; Meeprasert et al. 2023). Therefore, in addition to having antimicrobial activity, an irrigant solution must also be biocompatible (Martin et al. 2014) and, ideally, bioactive—capable of inducing osteo/odontogenic differentiation (Huang et al. 2008; Cassiano et al. 2022). The biological properties of Ca(OCl)_2_, such as cytotoxicity (Coaguila‐Llerena, Ochoa‐Rodríguez, et al. 2024) and cell migration (Blattes et al. 2017), have been previously investigated in different cell types. However, there is no available literature on its direct effects on AP tissue ex vivo or on AP target cells in vitro. The ex vivo model is particularly valuable, as it enables assessment of the biological effects of irrigants not in isolated cell cultures but within a tissue‐based approach, encompassing various cell types and the extracellular matrix.

This study aimed to evaluate the biological effects of Ca(OCl)_2_ in comparison with NaOCl by assessing morphological changes in AP tissue using an ex vivo model, and viability, proliferation, chemotaxis and osteogenic differentiation of hAPCs in vitro. The null hypothesis was that there would be no significant differences between Ca(OCl)_2_ and NaOCl in terms of their ex vivo effects on AP morphology or their in vitro effects on hAPC viability, proliferation, chemotaxis and osteogenic potential.

## 2 Materials and Methods

**FIGURE 1:**
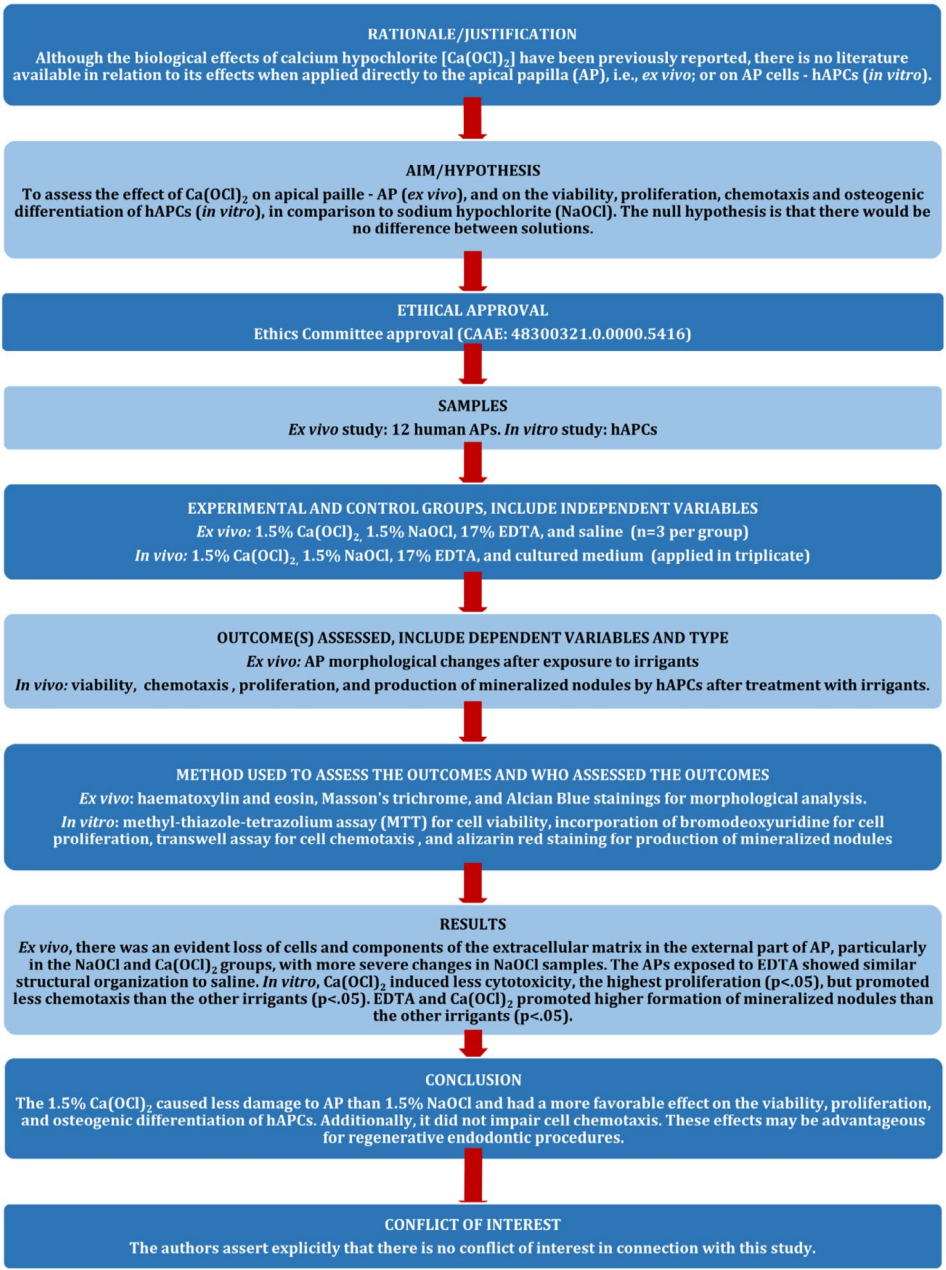
*Adapted from Nagendrababu et al. (2021). Full text available at: https://onlinelibrary.wiley.com/doi/abs/10.1111/iej.13542. For further details, visit: http://pridE‐endodonticguidelines.org/prile.

This manuscript was prepared in accordance with the Preferred Reporting Items for Laboratory Studies in Endodontology (PRILE) 2021 guidelines (Nagendrababu et al. 2021) (Figure 1).

### 2.1 Ex Vivo Study

Following approval from the Human Research Ethics Committee of the School of Dentistry at Araraquara of Sao Paulo State University—UNESP (CAAE: 48300321.0.0000.5416), human third molars without carious lesions or periodontal disease, and with incomplete rhizogenesis, were obtained from healthy patients aged 14–17 years. The teeth, extracted for orthodontic reasons, had less than two‐thirds of the root development (Widbiller et al. 2019). A total of twelve APs were collected (*n* = 3 per group). Each AP was carefully excised from the root using a no. 15 scalpel blade and immediately placed (randomly) into 2‐mL Eppendorf tubes containing one of the following solutions: 1.5% Ca(OCl)_2_ (Sigma‐Aldrich, St. Louis, MO, USA), 1.5% NaOCl (AraQuímica, Araraquara, SP, Brazil), 17% EDTA (Biodinâmica, Ibiporã, PR, Brazil), or sterile saline. Each sample was exposed to its respective solution for 3 min. EDTA and saline were used as control solutions. The Eppendorf tubes were labelled by a single operator using a code corresponding to the irrigant and were subsequently delivered to the blinded primary operator. Randomisation of the papillae into the groups was carried out using the online tool Random.org (https://www.random.org). Saline served as the negative control, given its lack of toxicity to connective tissue. The EDTA group was included because this solution is commonly used in REPs as a final irrigant during the first appointment and is sometimes used as a single irrigant in the second appointment (American Association of Endodontists—AAE 2021). The concentration of 1.5% for both NaOCl and Ca(OCl)_2_ was verified using the iodometric method (iodine/sodium thiosulfate titration). After 3 min of exposure, specimens were fixed in 10% buffered formaldehyde for 24 h, followed by standard histological processing and paraffin embedding. Serial 5‐μm‐thick sections were obtained and stained with haematoxylin and eosin (H&E) for assessing morphological changes, Masson's trichrome for collagen fibre visualisation, and Alcian Blue for glycosaminoglycan identification in the extracellular matrix of the AP. Qualitative morphological analysis was performed by a trained examiner blinded to group assignment, using an optical microscope (Olympus BX51, Tokyo, Japan).

### 2.2 In Vitro Study

This experiment was conducted using hAPCs previously characterised in our laboratory (Cassiano et al. 2022). Briefly, APs were obtained from three healthy donors and the hAPCs were isolated using the enzymatic digestion as previously described (Cassiano et al. 2022). Third‐passage cells were subjected to immunophenotypic characterisation by flow cytometry using the following mesenchymal stem cell markers: CD146, CD90, CD105, and CD73; and haematopoietic markers: CD45 and CD34, according to the manufacturer's instructions (BD Biosciences, Pharmingen, San Jose, CA, USA) (Cassiano et al. 2022). The hAPCs were cultured in α‐MEM (Sigma‐Aldrich) supplemented with 10% foetal bovine serum (FBS; Gibco/Invitrogen, Waltham, MS, USA) and 1% penicillin–streptomycin (10,000 U/mL penicillin, 10,000 μg/mL streptomycin; Sigma Aldrich) in an incubator maintained at 37°C, 95% humidity and 5% CO_2_. Cells from the third to sixth passages were used in the assays. After trypsinisation, the cells were seeded into 24‐ or 96‐well plates (Corning Inc., Corning, NY, USA) containing α‐MEM supplemented with 10% FBS and incubated for 24 h to allow adherence to the culture substrate. After this period, the culture medium was replaced, and cells were incubated with different proportions (v/v) of medium combined with 1.5% Ca(OCl)_2_, 1.5% NaOCl, 17% EDTA, or medium alone. All assays were performed in triplicate or quadruplicate and repeated independently three times. The sample size (n) for each in vitro assay was calculated using G*Power software (version 3.1.9.7 for Windows; Heinrich‐Heine University Düsseldorf) to ensure statistical power greater than 80%, with a significance level of 5%.

### 2.3 Cell Viability

After adhesion in 96‐well plates, hAPCs (1.5 × 10^4^ cells/well) were exposed for 24 h to the irrigants [diluted in culture medium (α‐MEM plus 1% FBS)], at concentrations of 0.025%, 0.05%, 0.1%, 0.2%, 0.4%, 0.8%, and 1.6% (v/v). Negative control cells were maintained in culture medium alone. At the end of the exposure period, 100 μL of a 1.0 mg/mL MTT solution (Sigma‐Aldrich) was added to each well, and the cells were incubated for an additional 3 h. The formazan crystals were solubilised by adding 100 μL of acidified isopropyl alcohol. Absorbance was measured at 570 nm using a spectrophotometer (Spectramax M, Molecular Devices). The percentage of cell viability was calculated relative to the absorbance of the negative control (culture medium), which was considered as 100%.

### 2.4 Cell Proliferation

Cell proliferation was assessed using the bromodeoxyuridine (BrdU) incorporation assay (ELISA, Roche, Heidelberg, Germany). hAPCs (1.5 × 10^4^ cells/well) were cultured in 96‐well plates for 24 h, and then exposed to 0.05%, 0.1%, 0.4%, and 0.8% (v/v) concentrations of each irrigant (selected based on MTT results), or to culture medium alone (negative control). BrdU solution (10 μL) was added to each well. After 24 h of exposure, the medium was removed and cells were fixed with 200 μL/well of fixer (FixDenat, Roche) for 30 min. After removing the fixer, cells were incubated with anti‐BrdU antibody (1:100, 100 μL per well) at room temperature for 120 min. Wells were then washed with 1:10 diluted washing solution, followed by the addition of 100 μL of substrate solution per well and incubation for 5 min at room temperature. Absorbance was measured at 370 nm using a spectrophotometer (Asys UVM 340, Biochrom, Cambridge, UK). The percentage of proliferation was calculated relative to the negative control (culture medium), considered as 100%.

### 2.5 Cell Chemotaxis

For chemotaxis evaluation, hAPCs (3 × 10^4^ cells/well) were seeded in α‐MEM with 1% FBS in the upper chambers of transwell inserts (8‐μm pore size; Corning). The lower chambers contained 600 μL of either α‐MEM with 10% FBS (positive control), α‐MEM with 1% FBS (negative control) or α‐MEM with 1% FBS plus the irrigant at 0.05% (v/v). Four transwells were used per group. After 24 h of incubation, cells were washed with phosphate‐buffered saline (PBS). Cells remaining in the upper chamber were removed with a cotton swab. Cells that migrated through the membrane to its underside were fixed in 4% paraformaldehyde and stained with 4′,6‐diamidino‐2‐phenylindole (DAPI; Life Technologies, Carlsbad, CA, USA). Five random fields per transwell were photographed using a fluorescence microscope (EVOS Fl, AMC, Bothell, WA, USA), and cell nuclei were quantified using *ImageJ* software (National Institute of Health—NIH, Bethesda, MD, USA).

### 2.6 Production of Mineralised Nodules

Alizarin red staining (ARS) was used to evaluate the production of mineralised nodules. The hAPCs (1 × 10^4^ cells/well) were seeded in 24‐well plates. Osteogenic culture medium [α‐MEM supplemented with 0.2 mM L‐ascorbic acid (A8960, Sigma Aldrich) and 4 mM β‐glycerophosphate (50 020, Sigma‐Aldrich)], with irrigants at 0.00075% and 0.0015% (v/v), or without irrigants, was renewed every 2 days for 14 days. These dilutions were selected based on previous results showing no cytotoxicity in hAPCs exposed for 48 h and 14 days, since only viable cells are capable of producing mineralised nodules (Coaguila‐Llerena, Ochoa‐Rodríguez, et al. 2024). At the end of the experimental period (14 days), cells were fixed in 70% ethanol at 4°C for 1 h, rinsed with deionised water, and stained with 2% ARS (pH 4.2, Sigma–Aldrich) for 20 min. Mineralised nodules were then solubilised in 10% cetylpyridinium chloride (Sigma–Aldrich) under gentle agitation for 15 min, and the optical density was measured at 562 nm using a spectrophotometer (Asys UVM 340, Biochrom). Culture medium without the osteogenic supplements was used as a negative control for the assay. Results from the positive control (osteogenic medium) were considered as 100%.

### 2.7 Statistical Analysis

Statistical analysis was performed using GraphPad Prism, version 9.3.0 (GraphPad Software, La Jolla, CA, USA), with α = 0.05. Normality and homogeneity of variances were assessed using the Shapiro–Wilk and Brown–Forsythe tests, respectively. Only the MTT assay satisfied both assumptions and was analysed using two‐way ANOVA followed by Tukey's post hoc test. ARS and cell proliferation data followed a normal distribution but did not meet the assumption of homogeneity of variances; nonetheless, they were also analysed using two‐way ANOVA with Tukey's post hoc test, considering the robustness of ANOVA to moderate variance heterogeneity when group sizes are balanced (Field 2013). Chemotaxis data did not meet the assumption of variance homogeneity and were therefore analysed using the Kruskal–Wallis test followed by Dunn's post hoc test.

## 3 Results

### 3.1 Ex Vivo Study

**FIGURE 2:**
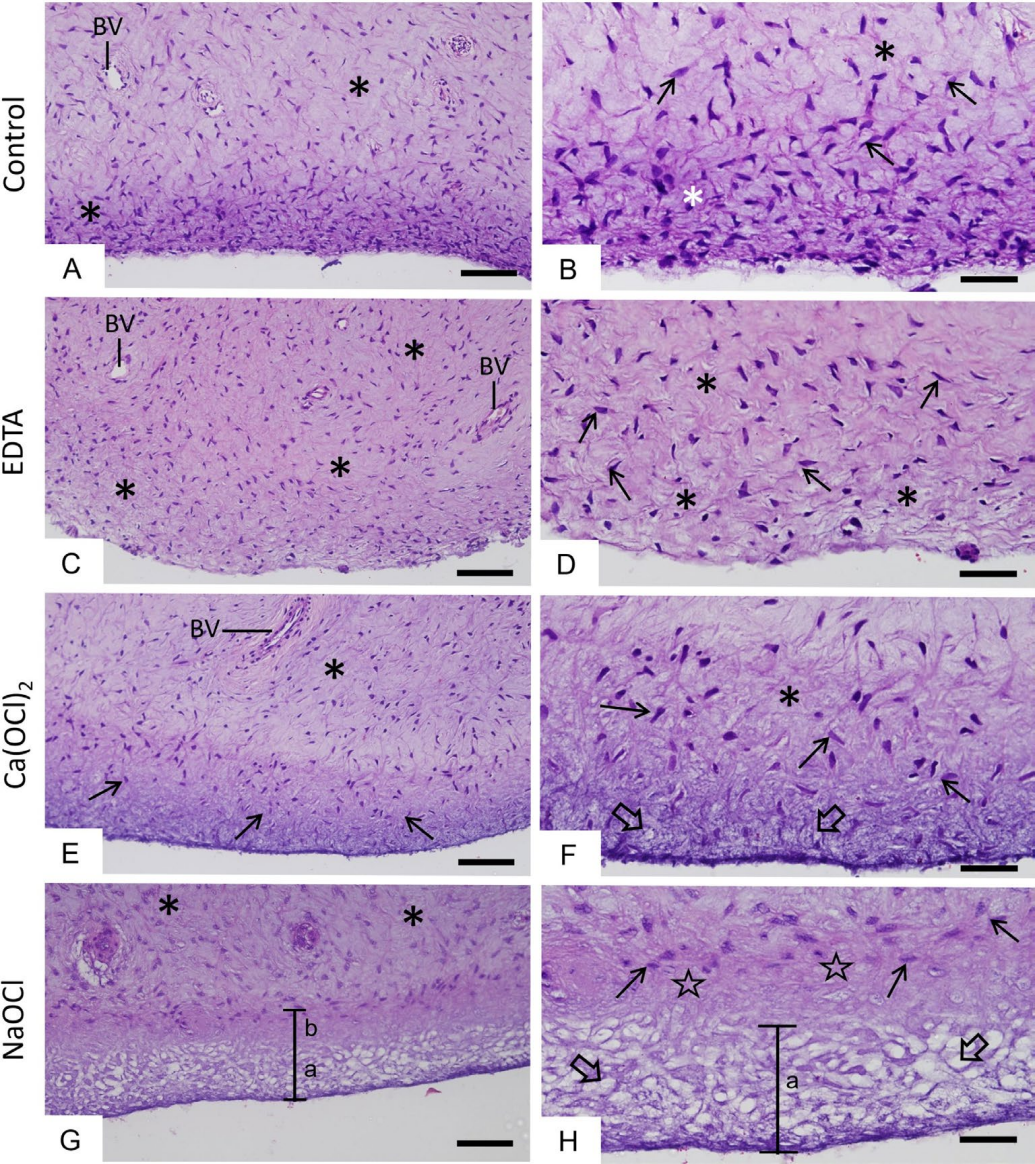
(A, B) Light micrographs of human apical papilla (AP) sections after treatment with saline, (C, D) 17% ethylenediaminetetraacetic acid (EDTA), (E, F) 1.5% calcium hypochlorite [Ca(OCl)_2_], and (G, H) 1.5% sodium hypochlorite (NaOCl). (A–D) Several fibroblasts (arrows) surrounded by dense extracellular matrix (asterisks) are observed throughout the connective tissue. (E, F) Few fibroblasts (arrows) are present in the outer layer of the papilla. At higher magnification (F), the extracellular matrix shows unstained areas (large arrows). (G, H) In the outer papilla layer (highlighted in G), two distinct regions are visible: In ‘a’, several unstained areas in the extracellular matrix (large arrows), in ‘b’, extracellular matrix is intensely stained (stars) and fibroblasts are scarce (arrows). BV: Blood vessel; H&E: Haematoxylin and eosin stain. Scale bars: 100 μm (A, C, E, G); 50 μm (B, D, F, H).

**FIGURE 3:**
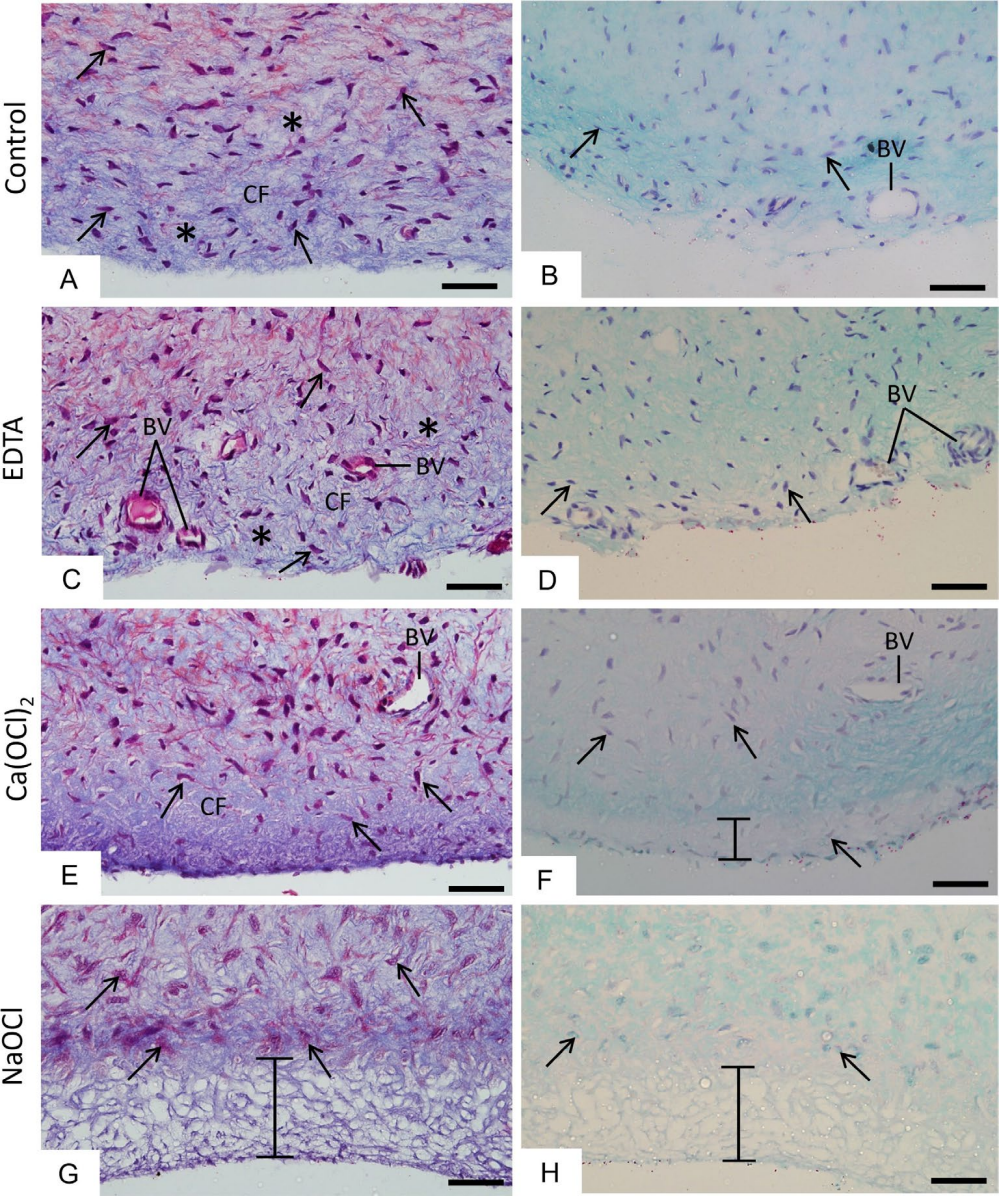
(A, B) Light micrographs of human apical papilla (AP) sections after treatment with saline, (C, D) 17% ethylenediaminetetraacetic acid (EDTA), (E, F) 1.5% calcium hypochlorite [Ca(OCl)_2_] and (G, H) 1.5% sodium hypochlorite (NaOCl). (A–D) Sections stained with Masson's trichrome. In (A, C), the extracellular matrix exhibits predominantly blue‐stained collagen fibres (asterisks) and numerous fibroblasts (collagen fibres—CF). (E) A dense bundle of blue‐stained collagen fibres intermingled with few fibroblasts (arrows) is observed in the outer layer of the apical papilla. (G) Absence of fibroblasts and scarce collagen fibres in the delimited outer apical papilla layer. B‐D‐F‐H: Sections stained with Alcian Blue (pH 2.5) and counterstained with haematoxylin. (B, D) Extracellular matrix shows homogenous staining. (F, H) Outer papilla layer (outlined region) shows (F) Alcian Blue‐negative or (H) weakly stained collapsed masses. BV, blood vessel. Scale bars: 50 μm.

APs from the saline group exhibited fusiform cells irregularly distributed within a surrounding extracellular matrix (Figures 2A,B and 3A,B). Specimens showed a peripheral layer with extracellular matrix intensely stained by haematoxylin (Figure 2B), blue‐stained collagen fibres (Figure 3A), and amorphous material reactive to Alcian Blue (Figure 3B). Following EDTA immersion, APs presented histological characteristics similar to those of the saline group (Figures 2C,D and 3C,D). In APs treated with 1.5% Ca(OCl)_2_, only a few fibroblasts were present in the outer layer (Figure 2E,F), with some unstained areas in the extracellular matrix (Figure 2F) or granular blue‐stained deposits (Figure 3E). Histochemical staining showed negative Alcian Blue reactivity in the outer layer of these specimens (Figure 3F). In contrast, APs exposed to 1.5% NaOCl displayed marked cellular and extracellular matrix alterations in the outer layer (Figures 2G,H and 3G,H), including a thicker band of collapsed, weakly stained material by H&E (Figure 2G,H) Masson's trichrome (Figure 3G), and Alcian Blue (Figure 3H), suggesting extensive matrix dissolution. Neither fibroblasts nor blood vessels were observed in the outermost layer of NaOCl‐treated APs, confirming the toxic effects of NaOCl (Figures 2G,H and 3G,H).

### 3.2 In Vitro Study

**FIGURE 4:**
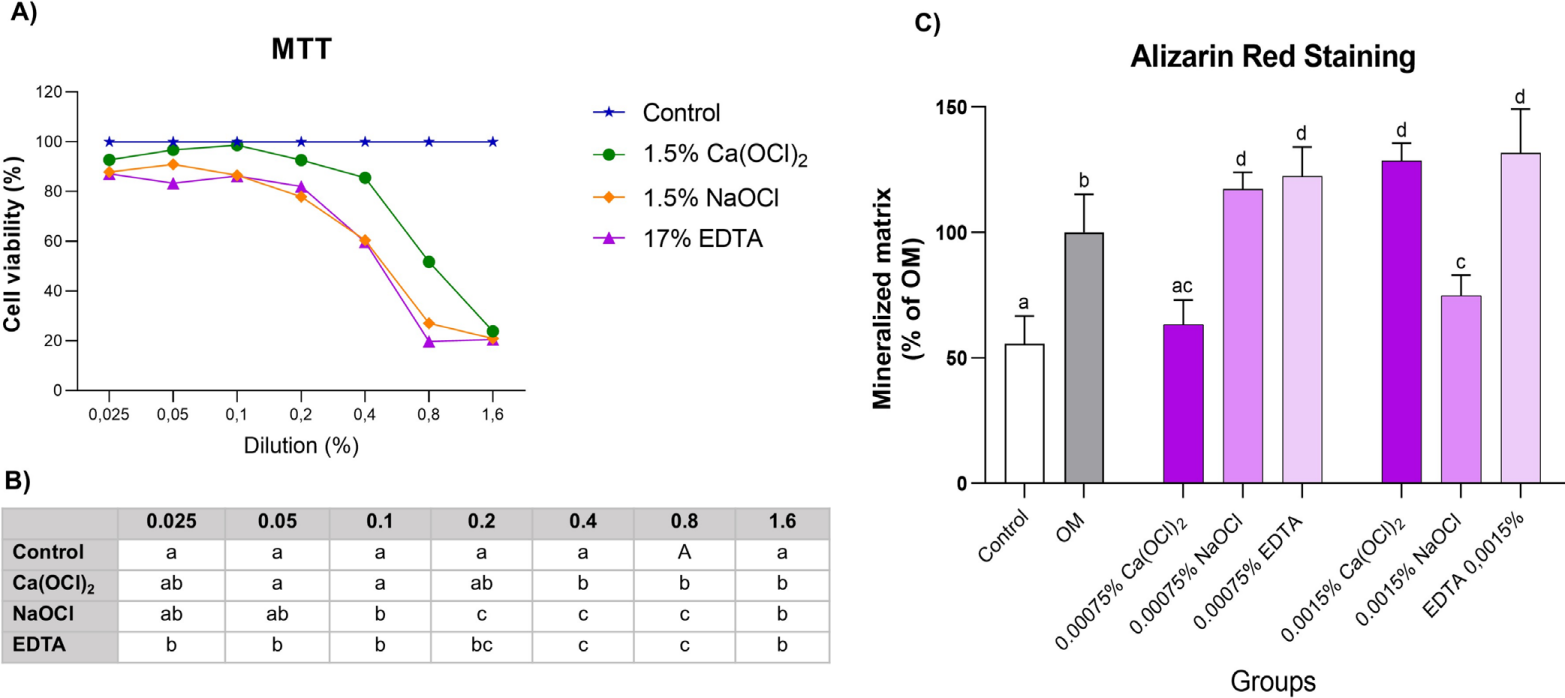
(A, B) Cell viability by methyl‐thiazole‐tetrazolium (MTT) assay. Viability of human apical papilla cells (hAPCs) after 24‐h exposure to various dilutions of 17% ethylenediaminetetraacetic acid (EDTA), 1.5% sodium hypochlorite (NaOCl), 1.5% calcium hypochlorite [Ca(OCl)_2_], for 24 h or α‐MEM with 1% foetal bovine serum (FBS) (control). (B): Different letters indicate statistically significant differences among groups (*p* < 0.05). *(C) Alizarin red staining assay. Comparison of mineralised nodule production among groups. Different letters above columns indicate statistically significant differences among groups (*p* < 0.05). OM, Osteogenic medium; control, non‐osteogenic medium.

**FIGURE 5:**
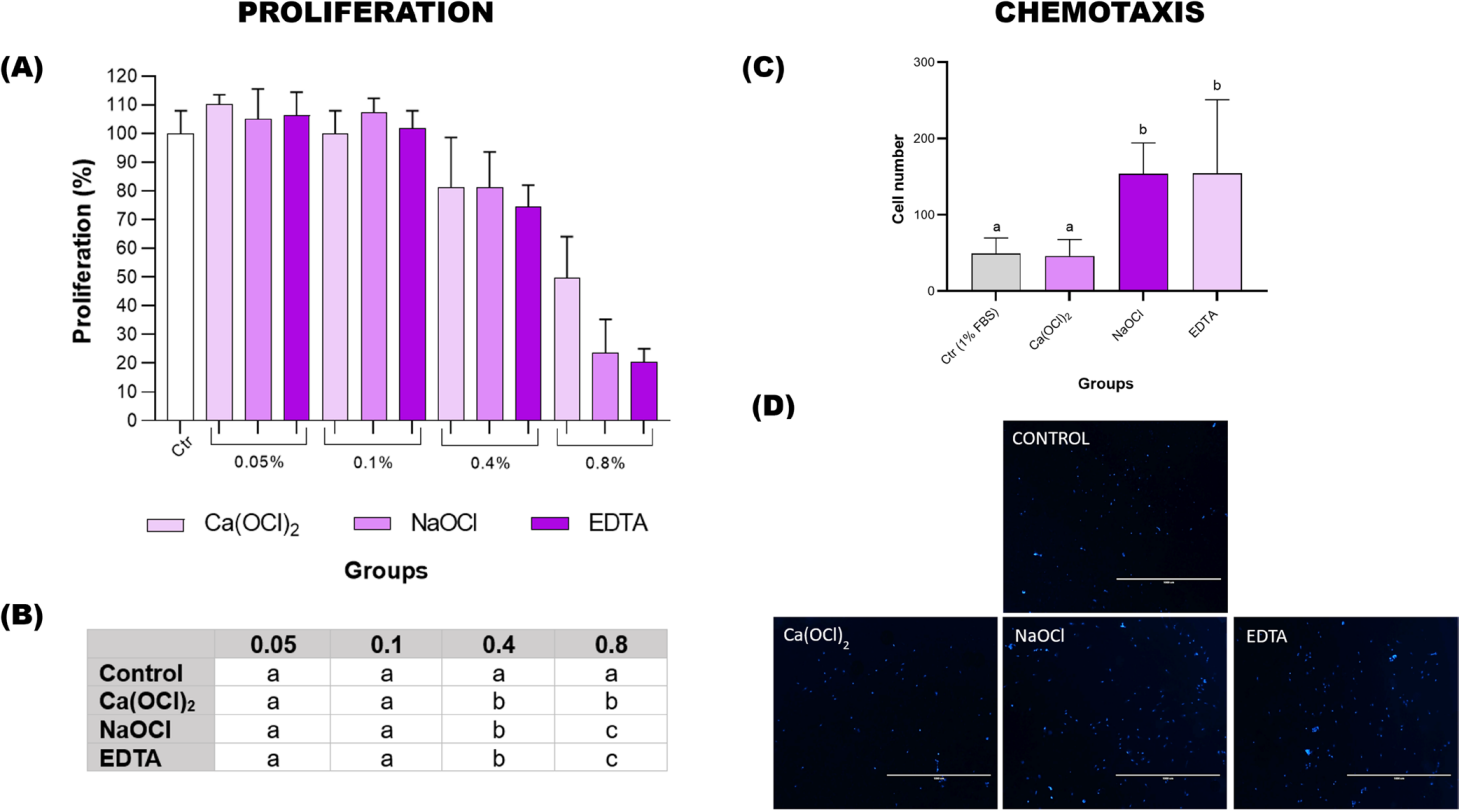
Cell proliferation and chemotaxis assays. (A) Proliferation of human apical papilla cells (hAPCs) by bromodeoxyuridine (BrdU) incorporation assay after 24 h exposure to culture medium (control), 17% ethylenediaminetetraacetic acid (EDTA), 1.5% sodium hypochlorite (NaOCl) and 1.5% calcium hypochlorite [Ca(OCl)_2_] at dilutions of 0.05%, 0.1%, 0.4% and 0.8% (v/v). (B) Statistical comparison of proliferation results. Different letters indicate significant differences among groups at each concentration. (C) Chemotaxis of hAPCs after exposure to 0.05% EDTA, NaOCl and Ca(OCl)_2_, or to control culture medium (CTR), assessed by Transwell assay. Different letters above columns indicate statistically significant differences among irrigants. (D) Representative images of hAPCs that migrated through the membrane. Cells were stained with 4′,6‐diamidino‐2‐phenylindole (DAPI) and visualised under a fluorescence microscope. Bar = 1000 μm.

Ca(OCl)_2_ was significantly less cytotoxic than both NaOCl and EDTA (*p* < 0.05), which showed comparable cytotoxic profiles (*p* > 0.05) (Figure 4). No significant difference in chemotaxis was observed between Ca(OCl)_2_ and the negative control (culture medium) (*p* > 0.05). By contrast, both NaOCl and EDTA significantly induced chemotaxis (*p* < 0.05) (Figure 5). Cell proliferation declined with increasing irrigant concentrations relative to the negative control (culture medium only) (*p* < 0.05). Notably, at the lowest concentration, Ca(OCl)_2_ induced greater proliferation than the other irrigants (*p* < 0.05), indicating better biocompatibility (Figure 5). In the mineralisation assay, EDTA and NaOCl induced more mineralised nodules at the higher dilution (0.00075%, v/v) than Ca(OCl)_2_ (*p* < 0.05). Nevertheless, Ca(OCl)_2_ still promoted more mineralised nodule formation than the control (culture medium) (*p* < 0.05), indicating its osteogenic potential even at lower concentrations. At the lower dilution (0.0015%, v/v), both EDTA and Ca(OCl)_2_ induced significantly more mineralised nodules than NaOCl (*p* < 0.05), further supporting the greater cytotoxicity of NaOCl (Figure 4).

## 4 Discussion

This study aimed to assess the effects of Ca(OCl)_2_ on AP tissue using an ex vivo model. In parallel, its effects on the viability, proliferation, chemotaxis and osteogenic differentiation of hAPCs were investigated in vitro, in comparison with NaOCl. The null hypothesis was rejected, since significant differences were observed between Ca(OCl)_2_ and NaOCl.

In the ex vivo study, human AP were used in a tissue culture model. The AP is situated apical to the root apex, separate from the dental pulp, and is responsible for the progressive elongation and maturation of the root. Within its collagen‐rich matrix, the AP contains a variety of cell types and a high concentration of stem cells (Driesen et al. 2021). Although the absence of blood supply and lymphatic drainage represents a limitation, the ex vivo assay model enabled the evaluation of irrigant effects on AP tissue, allowing interactions among different cell types and the surrounding matrix. This provides a relevant alternative to animal models, with the added benefit of using human tissues and cells (Nikolić et al. 2019).

According to the European Society of Endodontology (ESE) and the American Association of Endodontists (AAE), NaOCl concentrations for REPs may range between 1.5% and 3% for 5 min (Galler, Krastl, et al. 2016; American Association of Endodontists (AAE) 2021). In the present study, the NaOCl concentration was 1.5%, representing the lowest value within this recommended range, while the exposure time of all solutions was standardised at 3 min. This reduction in exposure time was justified by findings from our pilot study, which showed exposure to 1.5% NaOCl for 5 min resulted in almost complete AP dissolution.

To our knowledge, this is the first study to demonstrate the ex vivo impact of direct AP exposure to irrigating solutions. H&E staining was used to assess the morphological changes, Masson's trichrome to visualise collagen fibres (Pascon et al. 1991), and Alcian Blue to observe connective tissue glycosaminoglycans in the extracellular matrix of the AP (Calabrese et al. 2017; Scott 1996). APs exposed to 17% EDTA presented a histological appearance similar to that of the saline‐treated group. In contrast, APs treated with 1.5% Ca(OCl)_2_ exhibited less tissue destruction and less damage to the collagen fibres and extracellular matrix than those treated with 1.5% NaOCl. Although direct comparisons with prior studies are limited, it is known that 4.65% NaOCl and 5% Ca(OCl)_2_ can both promote soft tissue dissolution, although the effect is more pronounced with NaOCl (Dutta and Saunders 2012; Claudino Ribeiro et al. 2022). In ex vivo models, 5.25% NaOCl has also been shown to degrade bone marrow and compromise the overall density and structural integrity of mineralised bone tissue (Kerbl et al. 2012). Tissue dissolution has been reported to begin at concentrations as low as 1.5% of NaOCl (Claudino Ribeiro et al. 2022). Given that 1.5% Ca(OCl)_2_ resulted in less tissue destruction than 1.5% NaOCl, its effects on processes related to tissue repair were subsequently assessed in vitro using hAPCs. The AP is known to contain abundant mesenchymal stem cells (Sonoyama et al. 2006; Huang et al. 2008), as confirmed by immunophenotyping, which showed expression of CD146, CD90, CD105, and CD73 (Cassiano et al. 2022).

Ca(OCl)_2_ at 1.5% was less cytotoxic to hAPCs than 1.5% NaOCl and 17% EDTA after 24 h of exposure. This finding is supported by previous research in L929 fibroblasts, which indicated that although Ca(OCl)_2_ and NaOCl share similar cytotoxic mechanisms, the effects of Ca(OCl)_2_ are milder (Coaguila‐Llerena, Ochoa‐Rodríguez, et al. 2024), as corroborated by the present study. Other studies have similarly reported the lower cytotoxicity of Ca(OCl)_2_ in comparison with NaOCl in cell lines such as L929 (Sedigh‐Shams et al. 2016; Coaguila‐Llerena et al. 2019) and in human periodontal ligament cells (Coaguila‐Llerena et al. 2019). However, 1.5% NaOCl and 17% EDTA demonstrated comparable cytotoxicity in hAPCs. These findings diverge from those of Cassiano et al. (2022), who reported greater cytotoxicity of NaOCl compared to EDTA in hAPCs, possibly due to the higher NaOCl concentration used in their study (2.5% vs. 1.5% in the present investigation).

Cell proliferation plays a critical role in REPs, since it ensures a pool of cells capable of differentiating into specialised cell types, supporting tissue formation and facilitating vascular network development, which is essential to maintain cellular metabolism (Galler et al. 2015). In the present study, no significant differences were observed among the irrigants and the negative control (culture medium alone) at lower concentrations (0.05% and 0.1%, v/v). This finding contrasts with a previous study using the same BrdU incorporation assay and the same cell type (hAPCs), in which EDTA induced higher proliferation than NaOCl (Cassiano et al. 2022). However, that study used 2.5% NaOCl, whereas the present study tested 1.5% NaOCl. In a separate investigation using the MTT assay, no difference in hAPC proliferation was observed between 17% EDTA‐treated dentine and saline (negative control) after 24 h (Meeprasert et al. 2023). In the present study, Ca(OCl)_2_ induced higher proliferation than the other irrigants when used at the highest concentration (0.8% v/v).

Cell chemotaxis enables cells to reach the appropriate locations where they can receive essential signals for differentiation and effective tissue repair (Roca‐Cusachs et al. 2013; Galler, Widbiller, et al. 2016). In the present study, EDTA and NaOCl promoted greater chemotaxis than Ca(OCl)_2_. This finding contrasts with a previous study in which Ca(OCl)_2_ elicited higher cell migration than NaOCl. However, that study employed a different cell type (3 T3 fibroblasts), a lower concentration (0.00075%), and a different assay (scratch test) (Blattes et al. 2017). It is important to emphasise that, in the present study, Ca(OCl)_2_ did not impair chemotaxis, since no significant differences were observed in comparison with the negative control (culture medium with 1% FBS). In vivo, chemotaxis is influenced by numerous biomolecules, including chemokines and growth factors released during the healing process. Therefore, the lack of a chemotactic effect from Ca(OCl)_2_ should not be interpreted as a negative outcome. Nevertheless, this is an intriguing finding, considering that calcium ions can exert paracrine chemotactic effects and that both EDTA and NaOCl were more cytotoxic.

The presence of calcium ions in Ca(OCl)_2_ solutions (Coaguila‐Llerena et al. 2022) may contribute to osteogenic differentiation. At higher concentrations (0.0015%, v/v), Ca(OCl)_2_ induced more mineralised nodules than at the lower concentration (0.00075%, v/v), and even surpassed the positive control (osteogenic medium). Calcium ions are known to participate in mineralisation processes, such as the osteogenic differentiation of osteoblast precursors (Nakamura et al. 2010) or mesenchymal stem cells (Lee et al. 2018). Nevertheless, a previous study reported that the calcium ions in Ca(OCl)_2_ solution did not induce mineralised nodule formation in Saos‐2 cells (Coaguila‐Llerena, Ochoa‐Rodríguez, et al. 2024). This discrepancy may be explained by the fact that Saos‐2 cells are neoplastic and more committed to the osteoblastic phenotype than hAPCs.

Some limitations of the present study must be acknowledged. Although cell culture models are valuable for investigating cellular responses, direct extrapolation from hAPC cultures to in vivo conditions is not feasible due to the inherent complexity of the human organism (Hartung 2018). In the in vitro experiments, a dilution‐based approach was adopted to simulate clinical conditions in which irrigant solutions, aiming to replicate the in vivo situation, are unlikely to directly contact the mesenchymal cells located in the deeper regions of the apical papilla. It is also important to note that the dilution ratios of irrigants were selected according to the cytotoxicity observed for each outcome evaluated. Regarding the ex vivo study, although the histological effects of the irrigants on AP tissue were consistent across the three specimens per group, the small sample size (*n* = 3) represents a limitation. Additional studies, particularly clinical investigations, are warranted to validate and expand upon these findings.

Overall, the findings support the potential of Ca(OCl)_2_ as a viable irrigant for REPs, considering that it was less cytotoxic to both AP tissue and hAPCs, did not impair chemotaxis, and enhanced the proliferation and osteogenic differentiation of mesenchymal hAPCs.

## 5 Conclusion

The 1.5% Ca(OCl)_2_ solution caused less damage to AP tissue than 1.5% NaOCl and exerted more favourable effects on hAPC viability, proliferation and osteogenic differentiation. In addition, it did not impair cell chemotaxis. These findings suggest that Ca(OCl)_2_ may offer biological advantages as an irrigant for regenerative endodontic procedures.

## Author Contributions

Conceptualization: Gisele Faria, Paulo Sérgio Cerri, Carlos Rossa Júnior. Data curation: Hernán Coaguila‐Llerena, Gisele Faria, Bárbara Roma Mendes. Formal analysis: Gisele Faria, Paulo Sérgio Cerri, Hernán Coaguila‐Llerena, Bárbara Roma Mendes. Funding acquisition: Hernán Coaguila‐Llerena, Ellen Rabelo Ferraz, Bárbara Roma Mendes, Luana Raphael da Silva. Investigation: Hernán Coaguila‐Llerena, Gisele Faria. Methodology: Hernán Coaguila‐Llerena, Ellen Rabelo Ferraz, Bárbara Roma Mendes, Luana Raphael da Silva. Project administration: Gisele Faria. Resources: Gisele Faria, Paulo Sérgio Cerri. Software: Paulo Sérgio Cerri. Supervision: Gisele Faria, Paulo Sérgio Cerri, Carlos Rossa Júnior. Validation: Gisele Faria, Paulo Sérgio Cerri, Carlos Rossa Júnior. All authors drafted the manuscript. All authors contributed to reviewing and editing the manuscript. All authors have read and approved the final manuscript.

## Ethics Statement

The experiment was approved by the Ethics Committee of the School of Dentistry (CAAE: 48300321.0.0000.5416).

## Conflicts of Interest

The authors declare no conflicts of interest.

## Data Availability

The data that support the findings of this study are available from the corresponding author upon reasonable request.
